# Development and Evaluation of Polyvinyl Alcohol-Hydrogels as an Artificial Atrticular Cartilage for Orthopedic Implants

**DOI:** 10.3390/ma3042753

**Published:** 2010-04-14

**Authors:** Masanori Kobayashi, Hyon Suong Hyu

**Affiliations:** 1 Department of Biomedical Engineering, Daido University, 10-3 Takiharu-cho, Minami-ku, Nagoya, Aichi, 457-8530 Japan; 2 Department of Medical Simulation Engineering, Institute for Frontier Medical Sciences, Kyoto University, 53 Kawahara-cho, Shogoin, Sakyo-ku, Kyoto, 606-8507 Japan; E-Mail: biogen@frontier.kyoto-u.ac.jp (H.S.H.)

**Keywords:** polyvinyl alcohol-hydrogel, biocompatibility, artificial cartilage, orthopedics implant

## Abstract

Due to its excellent biocompatibility and mechanical properties, various different applications of polyvinyl alcohol-hydrogels (PVA-H) has been attempted in many fields. In the field of orthopedic surgery, we have been engaged for long time in research on the clinical applications of PVA-H as a artificial cartilage, and have performed many basic experiments on the mechanical properties, synthesis of PVA-H, and developed orthopedic implants using PVA-H. From these studies, many applications of artificial articular cartilage, intervertbral disc and artificial meniscus *etc*. have been developed. This review will present the overview of the applications and recent advances of PVA-H cartilages, and discuss clinical potential of PVA-H for orthopedics implant.

## 1. Introduction

Due to its excellent biocompatibility and mechanical properties, the bio-medical application of polyvinyl alcohol-hydrogels (PVA-H) is artificial arteries, artificial cartilages, and artificial muscles, *etc*. in various fields have been under study for a long time. However, until now, this PVA-H has not been used in specific clinical applications, despite the numerous studies.

Since 1985, Oka *et al*. have been engaged in research on the clinical application of PVA-H in the orthopedic surgery field, and have performed many basic experiments on PVA-H, and developed PVA-H implants. We also joined this research group and have been engaged in various research projects involving PVA-H. In this review, we will overview a series of findings we have discovered so far regarding the clinical applications of PVA-H for orthopedic surgery implants.

## 2. The Development of PVA-H

PVA was developed in 1924 by Hermann *et al*., and is a synthetic fiber with various excellent mechanical properties. It is also the raw material of “vinylon” the first high strength and high modulus synthetic fiber and PVA-H is prepared from this PVA. It is not only used for the manufacture of high strength high modulus fiber, but also serves as a raw material for films and acetal resins, as a textile processing agent, adhesive agent, polyvinyl chloride polymerization stabilizer, and inorganic binder, *etc*. Especially, since the excellent biocompatibility of PVA-H has been widely known, this has raised great expectations for its use as a biomaterial.

Generally, hydrogels were defined as gels which contain water but are not soluble in water, therefore, PVA-H was not also a strong gel, but it was turbid. Then, Hyon developed the freeze-throw method [1,2] and made a complete homogenous PVA solution by heating the mixture of PVA and water/dimethylsulfoxide (DMSO), after agitating under a nitrogen air current, leaving the solution in low temperature (−20 °C ) for 10 hrs, and promoting the crystallization and cross-linking of PVA molecules. This frozen gel was brought into contact with water to exchange DMSO in the PVA gel with water. Repeating this cryogels formation process by freeze-thaw cycling, a PVA-H with high mechanical strength, high water content, and excellent transparency was successfully produced.

By using this technique, we have developed the reinforcement of the mechanical properties of PVA-H by only the arrangement of water content and polymerization of PVA-H without any chemical agent or other blending polymers. Adding the cross-linking by γ-radiation, we have provided further improved mechanical properties and biocompatibility of PVA-H.

**Figure 1:**
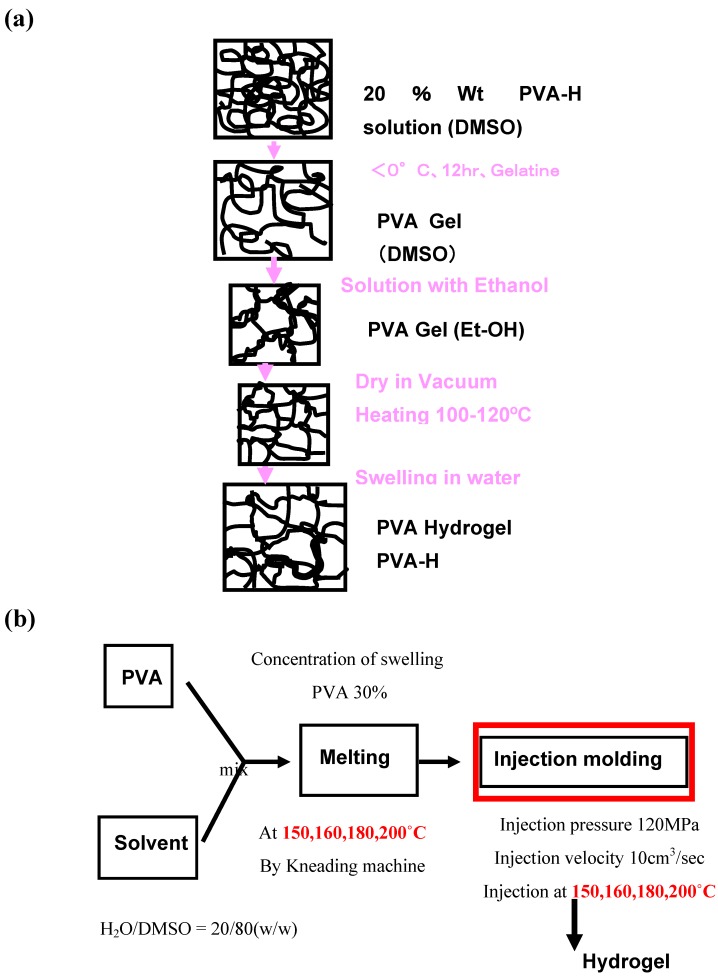
(a) Flow chart of the production process of Polyvinyl alcohol-hydrogel (PVA-H) by freeze-thaw method. (b) by injection molding method.

However, on the way to develop the PVA-H as an arthroplasty material, it was necessary for PVA-H to infiltrate into titanium fiber mesh as a composite devise to fix PVA-H firmly to the underlying bone. Because of poor infiltration of PVA solution into the pores of titanium mesh using the low temperature crystallization method, consequently, the filtration technique was improved by adopting high-pressure injection molding. The bonding strength of PVA-H and titanium fiber mesh interface was increased remarkably with this improved fabrication technique. Figure 1 shows the flow charts of the fabrication process of two types of PVA-H. After that, we have selected and examined two kinds of PVA-H, according to the medical device we tried to investigate.

## 3. Biocompatibility of PVA-H

It has already been reported that PVA-H has excellent biocompatibility, however, as we had not tested our newly improved PVA-H, so *de novo* basic biocompatibility experiments were performed using animals [3,4]. This PVA-H was implanted into various sites in rabbits and dogs, subcutaneously, intramuscularly, *etc*. and follow-up after surgery confirmed superior biocompatibility.

**Figure 2:**
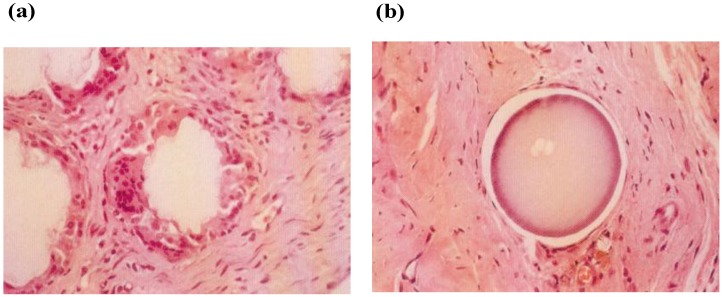
Histological appearance of the implant material particles and the surrounding tissue in rabbits knee joint. (×100) (a) UHMWPE particle: many macrophage and giant cells are surrounding particle due to intense foreign-body reactions. (b) PVA-H particle: foreign-body reactions were hardly observed.

In order to make certain *in vivo* immune-reactions against PVA-H, fine particles of PVA-H and ultra-high molecular weight polyethylene (UHMWPE), which is a common material for artificial joints, were injected into the bilateral knee joints, respectively, in the same rabbit and compared. The PVA-H used was fine particles with a diameter of 100 μm, with UHMWPE particles as control. Figure 2 is a histological photograph of tissue observed three months after injection. In the periphery of the UHMWPE particles, accumulated macrophages and foreign-body giant cells, and remarkable foreign-body reactions were observed, while, almost no reactions were seen around PVA gel particles on the opposite side. This bio-inert characteristic of this PVA-H is speculated to be due to the strong hydrophilic effects of this gel, which inhibits water circulation to the cells, and secondary, makes it difficult for cells to adhere to its surface.

The results of these basic experiments confirmed that PVA-H has favorable biocompatibility. In fact, even in our many animal experiment subsequently performed for the development of implants, no foreign-body reactions nor inflammation were ever observed for PVA-H.

## 4. Bio-Mechanical Properties of PVA-H

PVA-H has been chosen for investigation as it possesses several useful properties, including permeability, hydrophilicity and low frictional function. It has been widely commercialized and studied in the medical industries for the production of membranes, gels and films for an artificial pancreas, drug delivery system and adhesion protection sheet, *etc*. [5,6,7]. However, the use of PVA-H in the orthopedic surgery field has been thought to be limited because of its low mechanical strength and durability.

### 4.1. Mechanical properties of PVA-H

Actually, PVA-H was not so such a strong gel mechanically. Traditional cross-linking methods have been used to synthesize PVA-H materials with improved mechanical properties, however, the chemical agents introduced in their preparation are often toxic, inevitably affect the biocompatibility of the PVA-H. On the other hand, polymer blending is a useful method of improving or modifying the physiochemical properties of polymer materials, blends between synthetic polymers and biopolymers are of particular significance in current hydrogel research. A polymer blend can be defined as a combination of two polymers without any chemical bonding between them. Some chemical interaction can occur between components, but it sometimes induce the deterioration of mechanical properties of gels.

In this academic background, we succeeded in gelling PVA by cryogenic crystallization using a freeze-thawing method at low temperatures with the organic solvent DMSO as the synthesis process, making it possible to prepare PVA of improved transparency and high strength. Subsequently, the research and development of our orthopedic implants was performed using an improved version of this PVA-H, and it underwent further improvements such as molecule cross-linking by γ-irradiation in order to achieve higher strength.

On the way to developing the artificial cartilage, as mentioned above, it is necessary to infiltrate PVA-H into titanium fiber mesh as a composite device to fix PVA-H firmly to the underlying bone [4,8], so the high–pressure injection molding technique was used instead of the low temperature crystallization method. Accordingly, the mechanical properties of the PVA-H prepared by the new fabrication technique were subsequently investigated [9].

**Figure 3:**
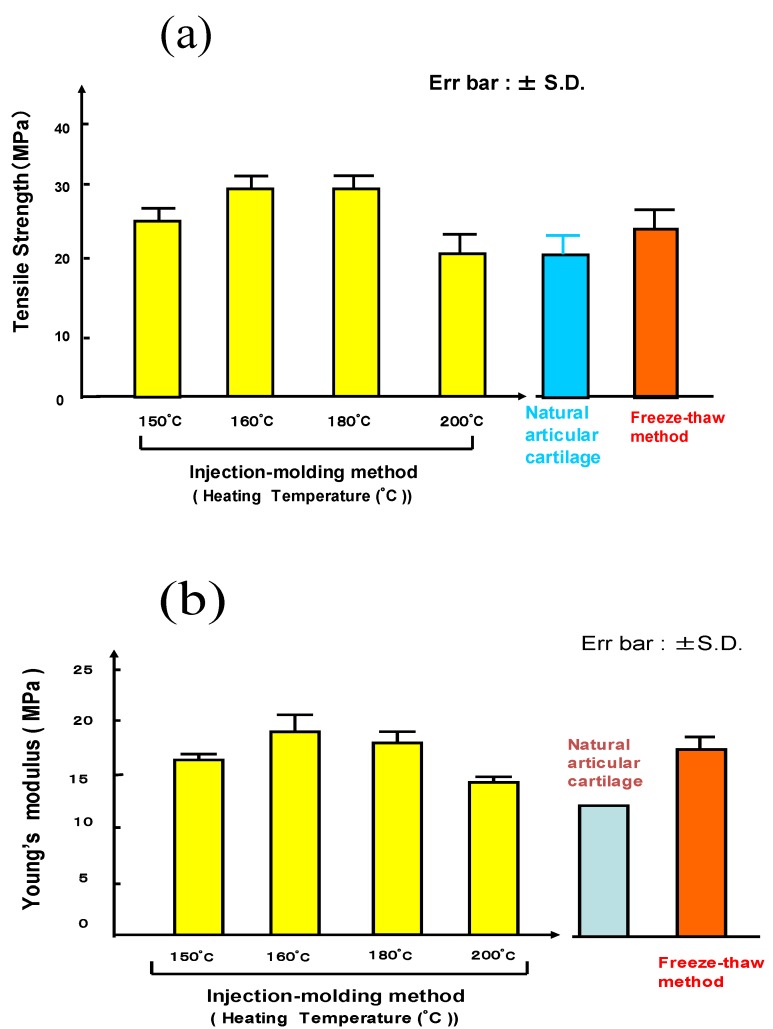
The mechanical properties of PVA-Hs by two fabrication methods (degree of polymerization: 8,800, Water content: 30%): (a) Influence of heating temperature on Young’s modulus (MPa). (b) Influence of heating temperature on ultimate tensile strength (MPa).

Figure 3 shows the tensile strength test results of PVA-Hs (degree of polymerization: 8,800, water content: 30%) prepared by two fabrication techniques. The results showed that the mechanical strength and Young’s modulus of gels did not correlate with the different freeze-thawing and injection molding fabrication methods, with regards to high PVA concentration.

**Figure 4:**
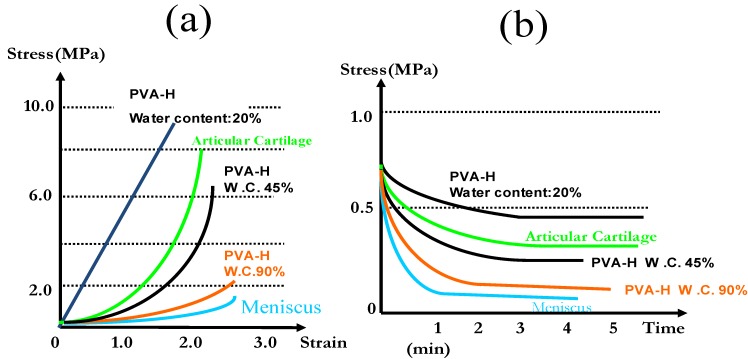
The visco-elastic characteristics of PVA-H. (a) Representative stress-strain curves of various PVA-H samples. (b) Comparison of the stress-relaxation curves of PVA-H samples.

The rheological behavior is also very important in investigating the mechanical properties of hydrogels. Although its variability depending on the measurement technique has been reported, we can evaluate the potentiality of its viscoelastic characteristics by comparing with that of natural human tissue [10]. Figure 4 shows the viscoelastic mechanical character of PVA-H. Figure 4(a) shows the stress-strain curves in compression tests of various PVA-Hs in some mechanical tests. As for the stress-strain curves of PVA-H, as seen from the curves, it has lower modulus values at the tangent in the primal phase of curves, which indicates a viscoelasticity typical of natural tissue such as a articular cartilage, exhibited in high water content gels, while, the elastic response (or solid-like response) dominated in low water content PVA-H. Figure 4(b) shows the results of stress-relaxation tests. PVA-H with a high water content tends to show more marked stress-relaxation. The human menisci exhibited the most acute stress-relaxation.

The results from mechanical testing indicated that changing the degree of cross-linking and water content could be utilized as a means of achieving the desired mechanical properties, including the viscoelastic characteristics of the PVA-H prepared by the freeze-thawing method or injection molding method.

### 4.2. Tribology of PVA-H

One of the important reasons that PVA-H has been widely studied as an artificial articular cartilage is its excellent lubrication function. Numerous authors have already reported the lubricative mechanism of PVA-H, which is said to be able to preserve the fluid film between articulating surface by elasto-hydrodynamic lubrication mode under certain loading condition. However, it was unclear if PVA-H under the boundary lubrication or solid lubrication mode could provide effective lubrication as a natural articular cartilage.

**Figure 5:**
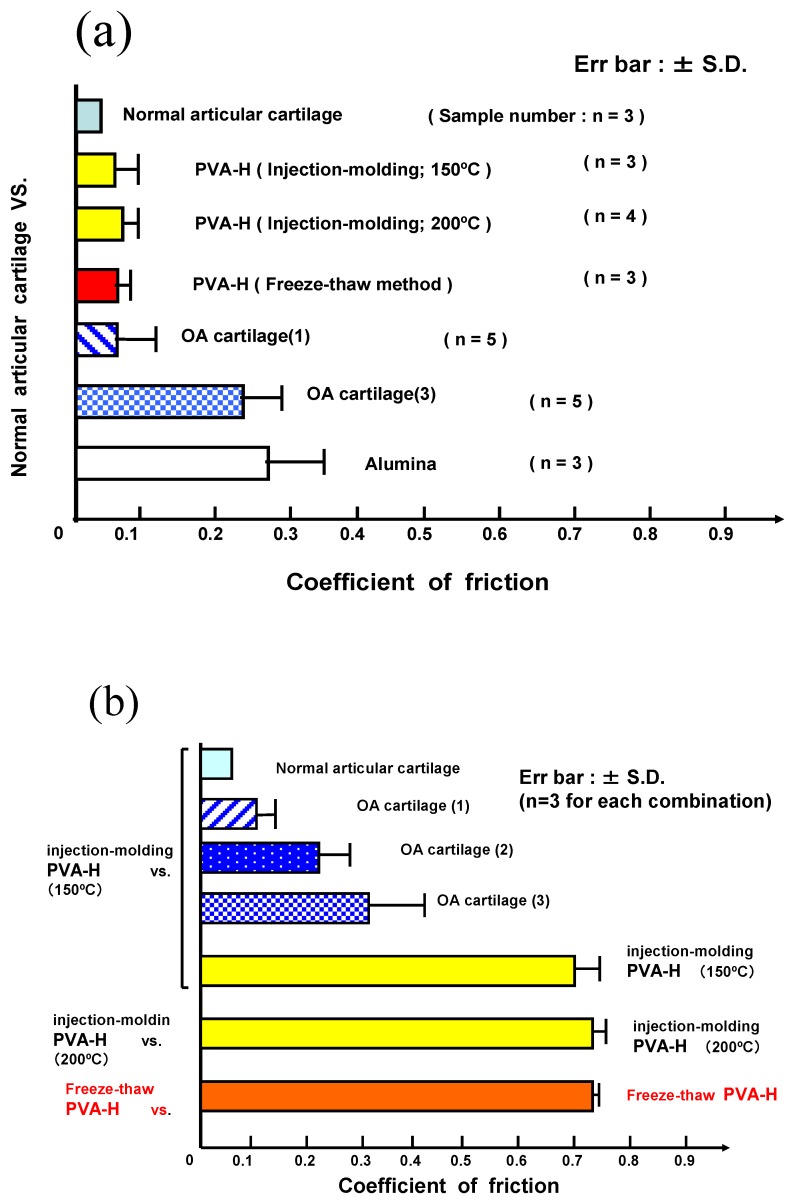
Coefficients of friction for various combinations of materials. Friction coefficients of various materials specimens against normal articular cartilage (a). against PVA-H(b).

The basic frictional function of PVA-H has been examined using an end face type friction test machine [10,11]. Figure 5 shows the frictional coefficient of various biomaterials against natural arrticular cartilage or PVA-H in synovial fluid simulated lubricants (saline with 0.375% hyaluronic acid, 3.0% albumin, 0.5% γ-globulin). All friction coefficients of PVA-H against articular cartilage showed low values compared to that of articular cartilage *vs.* articular cartilage, while the friction coefficients of PVA-H *vs.* PVA-H were remarkably high.

**Figure 6:**
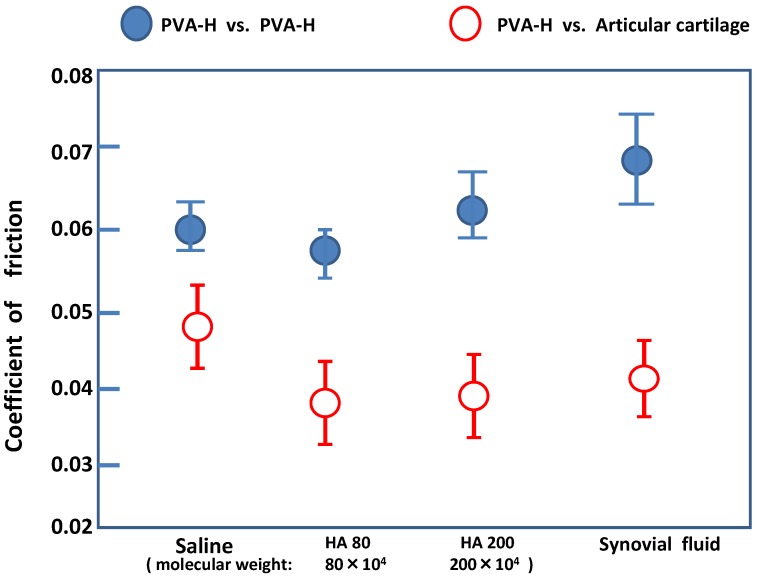
The influence of hyaluronic acid on the frictional coefficient of two combination of articular cartilage *vs.* PVA-H and articular cartilage *vs.* articular cartilage.

Figure 6 compares the friction coefficients of PVA-H *vs.* articular cartilage, and articular cartilage *vs.* articular cartilage with each different lubricant. Generally, the coefficient of PVA-H *vs.* articular cartilage was ineffective on the dose of hyaluronic acid in spite of the fact that the articular cartilages shows a hyaluronic acid dose dependence. These frictional experimental data means that the excellent lubricant mechanism of hyaluronic acid, including albumin and γ-globulin, on the articular cartilage surface could not operate on a PVA-H surface.

Therefore, as another experimental approach to examine the tribological function of PVA-H, we observed the behavior of surfaces of PVA-H and natural articular cartilage under high loading conditions by confocal laser scanning microscopy (CLSM) [12,13] .

**Figure 7:**
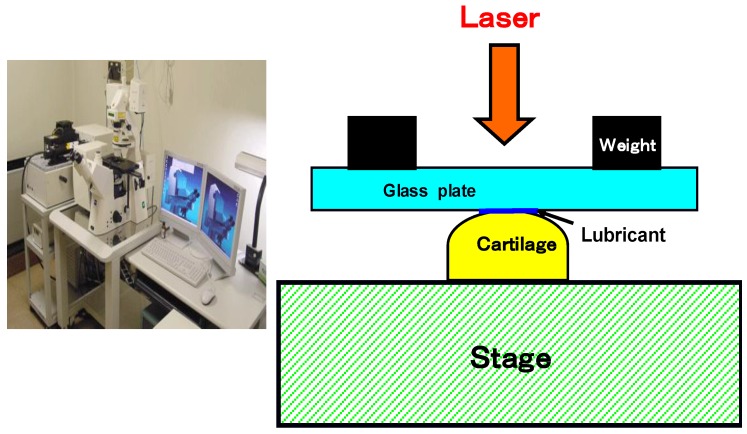
Photograph of real-time 1LM21 confocal laser scanning microscopy (CLSM) and schematic cross-section view of the CLSM observation.

Articular cartilage specimens of the normal knee joint (femoral condyle side) were obtained from adult rabbits weighting about 3 Kg. PVA-H with a water content 30%, a degree of polymerization 17,500 was prepared as a specimen by the freeze-thawing method. A ®1LM21 real-time CLSM (Lasertec. Co., Japan) instrument was used. Each specimen was placed on the table, the synovial fluid (lubricant) added to its surface, pressed with a glass plate (0.15 mm thickness) and a load (12 N) applied for CLSM observation (Figure 7).

**Figure 8:**
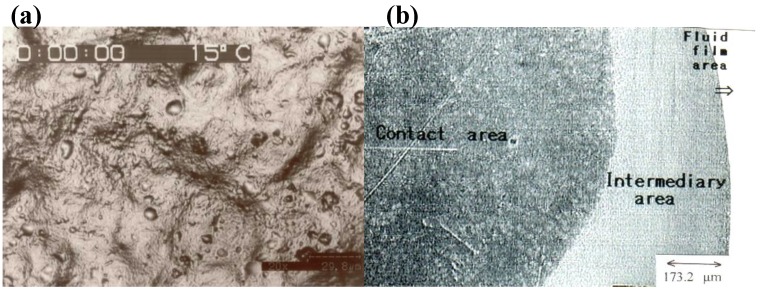
CLSM image of normal articular cartilage surface of rabbit femoral condyle of knee joint. (a) Normal articular cartilage surface has a lot of depressions and drops of the synovial fluid without loading. (×1,400). (b) CLSM image of normal articular cartilage surface under the loading (×250).

The compression of natural articular cartilage by a glass plate, which was equivalent to physiological loading, caused the cartilage surface to exhibit two distinct areas; one in direct contact area with glass plate and one with a fluid pool between the cartilage and the glass plate (Figure 8).

**Figure 9:**
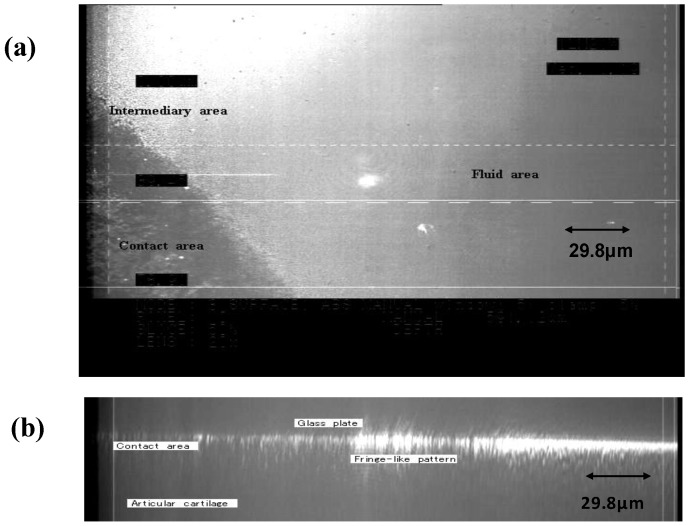
CLSM image of normal articular cartilage surface of rabbit knee joint under loading. (a) CLSM image of articular cartilage surface through glass plate (×1,000). (b) Cross-section view of CLSM image (×1,000).

**Figure 10:**
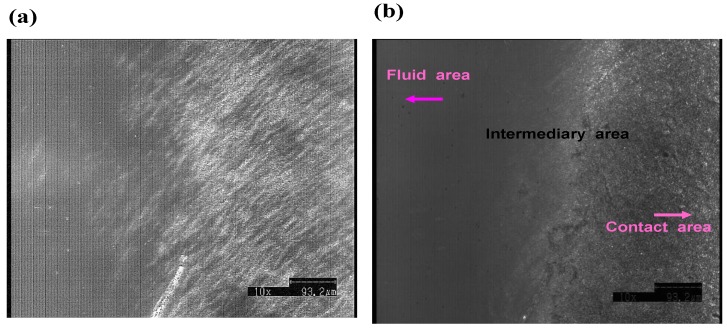
CLSM images with optical-isolator technique about the intermediary area. The reflection image (a) was obtained corresponding to the third area in the original CLSM image (b).

Between these two areas, a third morphological area was observed, as shown in Figure 9. In the area in direct contact with the glass plate, the detailed morphology in the articular cartilage surface disappeared due to compression. In the area of the fluid pool, the presence of a fluid membrane between the articular cartilage and the glass plate was confirmed, but the state of the cartilage surface is unclear. In the third area, a fringe-like pattern was observed around the contact area at higher magnification. In addition, the observation of this area by a particular optical-isolation method revealed a reflected image that corresponded to the third area, as shown Figure 10. This finding indicates that the third area is composed of a liquid crystal structure.

**Figure 11:**
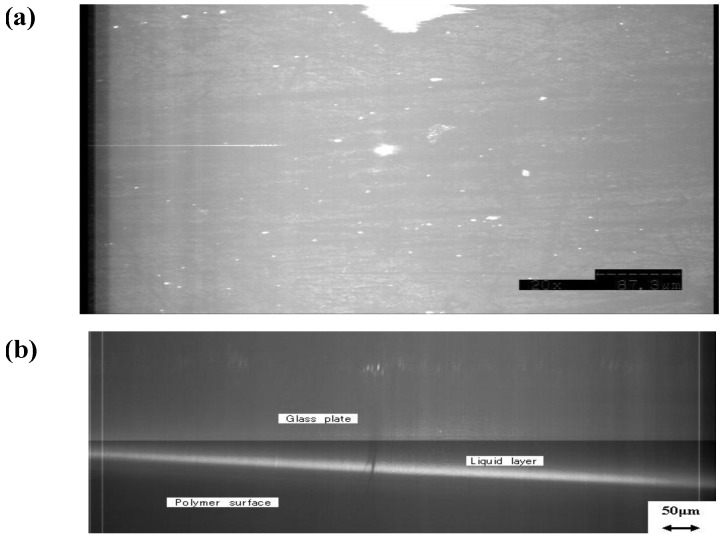
(a) CLSM image of PVA-H surface under the loading (×700). Third area and fluid pooling area were unclear. (b) Cross-section view of CLSM image (×700).

In contrast, the CLSM images of the PVA-H surface under loading were uniform, and distinguishing between the area of contact and the fluid pool was impossible, as shown in Figure 11 In the cross-sectional view, the grass plate and PVA-H surface were clear, but no images corresponding to the third area, which was seen in the articular cartilage specimens was observed. The optical-isolation technique also did not reveal any image.

To discuss the excellent lubricative mechanism of natural synovial joints under the high loading conditions such as boundary lubrication, we must address the molecular nature of the protective film layer that covers the articular cartilage surface. Many investigators have already proposed the presence of this lubricin [14,15,16]*.* Our CLSM image on articular cartilage may indicate that the liquid crystal region reflected the protective film layer on the cartilage surface. While PVA-H was developed to have the same lubricative mechanism of natural joints, however, CLSM of PVA-H did not reveal the presence of a film layer, though may demand further analysis and discussion, the finding means that PVA-H does not form a fluid film nor liquid crystal, as does natural articular cartilage, and PVA-H has limitations as an artificial cartilage in imitating the lubricative mechanism of synovial joint cartilage.

## 5. Application for Arthroplasty Implant

### 5.1. Development of artificial articular cartilage of PVA-H

Arthroplasty using implants as used in total hip joint arthroplasty (THA) and total knee joint arthroplasty (TKA), is an established treatment for patients with severe arthrosis caused by diseases such as osteoarthritis (OA) and rheumatoid arthritis (RA). However, the tolerance of artificial joints to physiological loading is inferior to that of natural joints, and the number of revision operations performed to adjust for loosening or wear of these implants has been increasing. The long term survival of THA and TKA is a significant problem in orthopedic surgery [17,18].

In contrast, it is known that natural joints have excellent lubrication. Though the mechanism involved is not still clear, triblogical studies, biomechanical studies and morphological studies have proposed various theories to clarify the mechanism behind the superior lubricative function of natural joints. If the mechanism of natural joint lubrication could be imitated by artificial materials, the properties of artificial joints could be much improved.

In this respect, we have attempted to develop an artificial articular cartilage based on PVA-H. However, our frictional test results of PVA-H exhibited high frictional coefficients and much wear, enough to predict the failure of arthroplasty by PVA-H. The use of PVA-H for total arthroplasty has a problem regarding the lubrication between two PVA-H surfaces.

### 5.2. Development of artificial articular cartilage as a hemi-arthroplasty

On the other hand, as mentioned before, the friction coefficients of PVA-H against normal articular cartilage have shown low values in frictional tests. This finding encouraged us to attempt to use PVA-H as a medical implant for partial (hemi-)arthroplasty replacement.

Osteonecrosis of the femoral head is a disabling disease that can lead to destruction of the hip joint. The appropriate treatment, depending on the stage of the disease, remains controversial, yet progression to collapse of the femoral head often necessitates total hip replacement (THR). Considering the age of patients and the poor prognosis associated with THR, it is desirable to preserve as much of the joint as possible during treatment as in the concept of an artificial cartilage for osteoarthritis [22,23,24].

**Figure 12:**
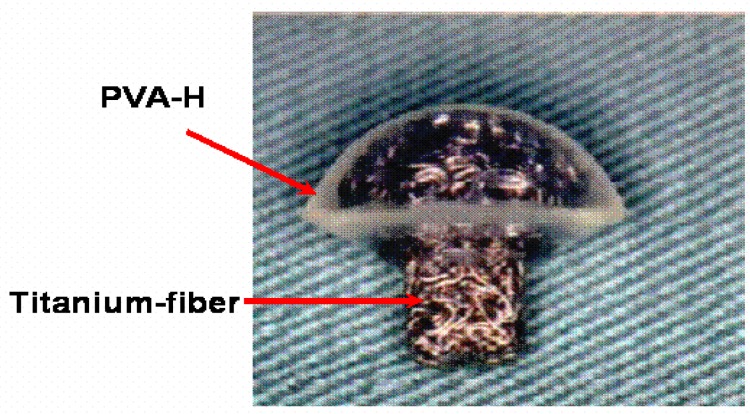
Photograph of a synthetic composite osteochondral device for partial replacement of the canine femoral head. The transparent portion is made of PVA-H.

We have developed an articular cartilage composite device with PVA-H whose mechanical properties and lubricating functions have been characterized, and a titanium fiber mesh which is intended to attach PVA-H firmly to underlying bone as shown in Figure 12 [25]. The PVA-H used was polymerization degree 8800, water content 30%, which corresponds to the mechanical properties of natural articular cartilage *in vitro*.

**Figure 13:**
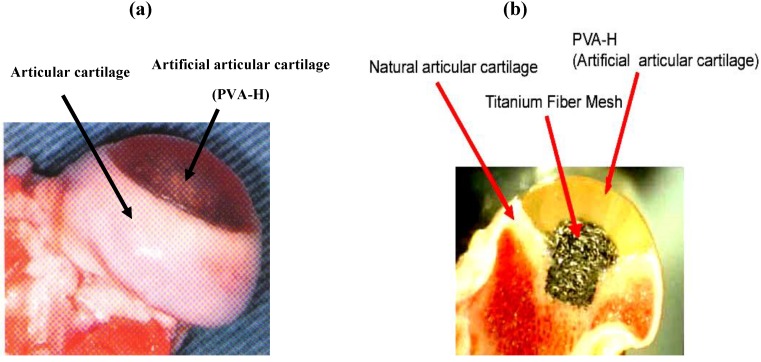
Photograph of a specimen of the canine femoral head with composite osteochondral device at 12 months after implant operation. (a) Macroscopic appearance. (b) Cross-sectional appearance.

This composite has been implanted into the surface of a canine femoral head for the purpose of simulating partial surface replacement hemi-arthroplasty, as shown in Figure 13. In addition to this PVA-H device, prostheses made from ultra-high-molecular-weight polyethylene (UHMWPE) and alumina ceramics were also implanted as a control group to compare with the present implant material.

The dogs with implant were allowed unrestrained weight-bearing and normal activity in their living area, and killed at time intervals of one, three, six, and 12 months. Dogs with all three types of devices showed good locomotive function of the hip joint without dislocation, deformity nor limping during their survival periods.

**Figure 14:**
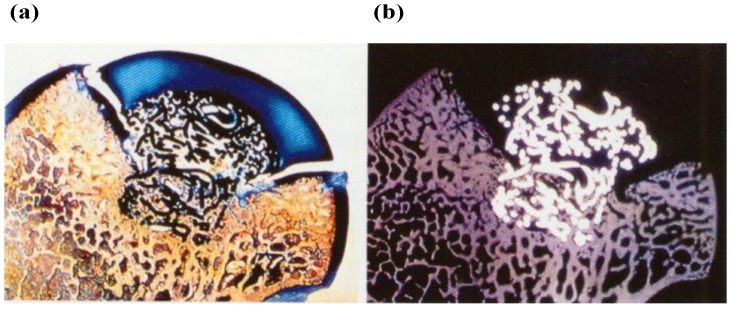
Cross-section view of a specimen of canine femoral head with a composite osteochondral device at 12 months after implant operation (×2.5). (a) Histological appearance (Giemusa stain ). (b) Contact microradiograph appearance.

Concerning the endurance of the PVA-H prosthesis, the transected specimens showed good congruity of the PVA-H with the adjacent natural articular cartilage. Figure 14 shows the histological and radiographic findings at 12 months after operation. It shows that the reduction and position of femoral head were well maintained, there was a increase in bone density around the stem portion of the titanium fiber mesh and good fixation to the surrounding bone.

To evaluate the lubrication function of the PVA-H prosthesis, opposite canine acetabular articular cartilages were also reviewed macroscopically and histologically. There were no adverse responses, such as erosion or hemorrhage observed in the macroscopic appearance of the articular cartilage of acetabulum of the PVA-H group, while there was slight synovitis and discoloration in the alumina group. In the UHMWPE group, there was severe synovitis and erosion of the acetabular cartilage six months after the operation.

**Figure 15:**
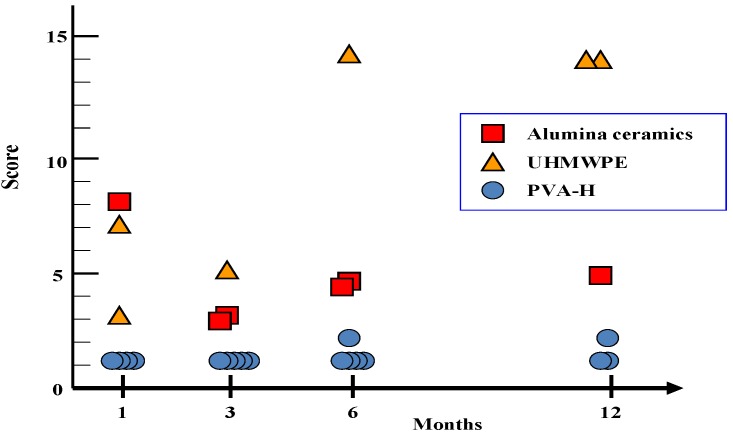
Mankin scores for the changes in acetabular cartilage in each group. Each point represents a single score for a joint.

Histological evaluation of acetabular articular cartilage was graded using the methods reported by Mankin *et al.* [26]. These results are summarized in Figure 15. The PVA-H group acetabular cartilage showed that structural integrity, surface regularity and thickness were well maintained throughout the experimental periods, while the histological appearance of the other material groups deteriorated depending on the experimental periods.

Although this study has limitations as an evaluation, we think these results demonstrate the superior potential of the partial surface replacement hemi-arthroplasty by PVA-H articular cartilage.

## 6. Current Problems and Clinical Potential of PVA-H Cartilage

### 6.1. Current problems of PVA-H

The greatest current problem regarding the clinical application of this PVA-H is the lack of a firm fixation method with living tissues. PVA-H has superior bio-inert properties, and hardly adheres or binds to the living body. As already mentioned, in order to couple these PVA-H implants with living bone we tried to conjugate them with titanium fiber mesh [27,28,29,30]. Other research reports have also already confirmed the ingrowth of newly formed bone in titanium mesh, and if this method proves to be favorable for the attachment between PVA-H and titanium mesh, its practical application can be expected. However, the conjugation of this PVA-H with the titanium mesh is first and foremost similar to machine parts, and there still remains incertitude regarding its long-term attachment *in vivo*.

On the other hand, as in recent years research on the bio-activation of material surfaces and the interaction between artificial material surfaces and living tissue has made great progress, the development of other adhesion techniques with natural tissue is greatly anticipated. As a result of a large amount of research, including the studies of scaffolds for tissue engineering, adhesion mechanisms between material surfaces and tissue cells already being gradually elucidated, techniques that enable the adhesion of living cells to biomaterials are being reported one after another [31,32]. Also regarding PVA-H, Hayami *et al*. have reported that by surface treatment adhesion of soft tissues is possible [33]. We also are considering methods for the bio-activation of this PVA-H gel surface.

### 6.2. Clinical potential of PVA-H cartilage

Although PVA-H cartilage still has some problems regarding the fixation method, the biocompatibility of PVA-H has been confirmed, as has the advantage for PVA-H to be able to adjust the appropriate mechanical properties as a substitute for the desired target tissues by adjusting the water content and the degree of polymerization. Actually, the some challenges regarding clinical orthopedics implants using PVA-H, aside from the articular cartilage, have been already tried, as shown in Figure 15 [34,35,36]. Furthermore, we have already succeed in the improvement of the mechanical properties of PVA-H by fabricating the compressive-orientation reinforced PVA-H. This new PVA-H has high mechanical strength and wear durability.

**Figure 16:**
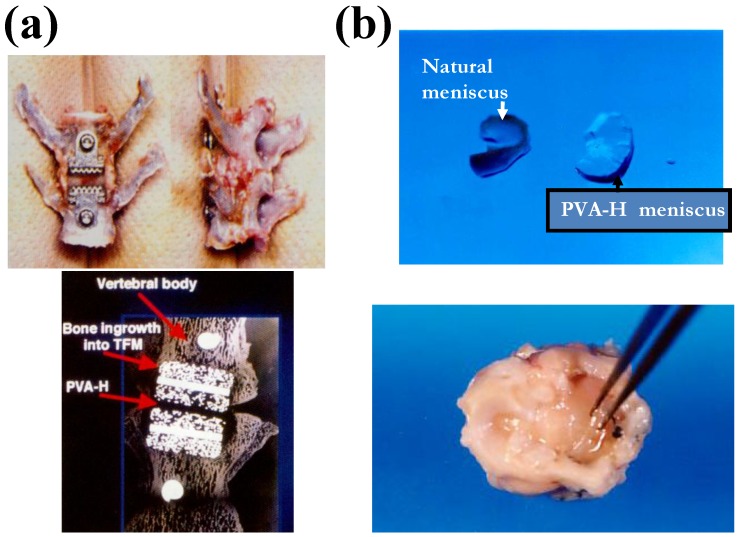
Other orthopedics implants using PVA-H. (a) Artificial intervertebral disc. (b) Artificial meniscus.

Considering another characteristics of PVA-H such as permeability, hydrophilicity and transparency *etc*., depending on ideas and ingenuity, PVA-H is an extremely promising material for various clinical applications including scaffold for tissue engineering. In the future together with the developments of medical technology, new methods for implant treatments with combined characteristics will be developed for each clinical field, and as far as PVA-H is concerned, it will be necessary to make an effort to attach new functions that are required based on the knowledge and techniques that were accumulated until now, also with an eye on clinically specific treatment methods.
